# Extracellular Vesicles in Multiple Sclerosis: Role in the Pathogenesis and Potential Usefulness as Biomarkers and Therapeutic Tools

**DOI:** 10.3390/cells10071733

**Published:** 2021-07-08

**Authors:** Marianna D’Anca, Chiara Fenoglio, Francesca Romana Buccellato, Caterina Visconte, Daniela Galimberti, Elio Scarpini

**Affiliations:** 1 Fondazione IRCCS Ca’ Granda, Ospedale Policlinico, 20122 Milan, Italy; caterina.visconte@gmail.com (C.V.); daniela.galimberti@unimi.it (D.G.); elio.scarpini@unimi.it (E.S.); 2 Department of Pathophysiology and Transplantation, University of Milan, 20122 Milan, Italy; chiara.fenoglio@unimi.it; 3 Department of Biomedical, Surgical and Dental Sciences, University of Milan, 20122 Milan, Italy; francesca.buccellato@unimi.it

**Keywords:** extracellular vesicles, microRNAs, biomarkers, multiple sclerosis, extracellular vesicles cargo, therapeutic approach

## Abstract

Although extracellular vesicles (EVs) were initially relegated to a waste disposal role, nowadays, they have gained multiple fundamental functions working as messengers in intercellular communication as well as exerting active roles in physiological and pathological processes. Accumulating evidence proves the involvement of EVs in many diseases, including those of the central nervous system (CNS), such as multiple sclerosis (MS). Indeed, these membrane-bound particles, produced in any type of cell, carry and release a vast range of bioactive molecules (nucleic acids, proteins, and lipids), conferring genotypic and phenotypic changes to the recipient cell. This means that not only EVs per se but their content, especially, could reveal new candidate disease biomarkers and/or therapeutic agents. This review is intended to provide an overview regarding current knowledge about EVs’ involvement in MS, analyzing the potential versatility of EVs as a new therapeutic tool and source of biomarkers.

## 1. Introduction

Extracellular vesicles were already known in 1967 when Wolf confused them for “platelet dust” [1], and for many years, EVs were considered exclusively as waste collectors. Their mere waste function has definitely been shelved since many fundamental biological roles in physiological and pathological conditions have been observed and addressed to EVs [2,3]. These membranous particles guarantee intercellular communication [4] thanks to an extraordinary variety of molecules inside them, made up of nucleic acids, proteins, and lipids. EVs’ content, termed as “cargo”, reflects the parental cells and could be poured into determinate target cells even more distant to the cell of origin [5]. The appealing characteristic of being able to cross the blood–brain barrier (BBB) from the brain to the blood and vice versa increased the interest in EVs in the neurological field too [6]. Thus, EVs’ involvement has been portrayed in the healthy and the primarily diseased central nervous system (CNS), such as neuroinfection [7], neuroinflammation [8], neurodegeneration [9], brain tumors [10], and psychiatric diseases [11]. In recent years, their significance in physiological aging is also burgeoning as linked to their multi-faceted implications in age-related diseases [12]. EVs secreted from different types of neural cells such as neurons, astrocytes, microglia, and oligodendrocytes keep track of parental cells through cell-specific receptors on their surface and with their cargo, becoming a reservoir of potential biomarkers [13]. In this way, detection of specific CNS-derived EVs, ideally in blood streaming, could represent a non-invasive way to remotely analyze the CNS status [14]. Even though MS was, more than 30 years ago, the first neurological disorder identified where the release of EVs is closely related to disease progression and neuroinflammation [15,16], research in this context is struggling to take off if compared to the oncologic field. In this line, EVs should deserve greater attention from the scientific community considering that they provide information on status pathogenesis and could serve, at the same time, as therapeutic targets and diagnostic tools [17,18]. This review aims to present an overview about EVs’ involvement in MS and to discuss the potential use of EVs’ cargo as a diagnostic biomarker and new therapeutic strategy.

## 2. EVs: Biogenesis, Composition, and Cargo Release

Based on the more recent definition of EVs, they are membrane-bound particles released by cells and unable to replicate [19]. The historical classification in exosomes and microvesicles based on their size and biogenesis route has been overcome. In 2018, to define EV subtypes, the Minimal Information for Studies of EVs (MISEV) guidelines, drafted by the International Society of EVs (ISEV), recommended to use more generic and operational terms referring to size (small, medium, and large EVs) or density (low-, mid-, and high-density EVs); biochemical composition (CD63+-, CD81+-EVs, etc.); and conditions or cell of origin (hypoxic, endothelial, neuronal EVs, etc.). 

Virtually, all cells are able to produce and release EVs that can exert their action locally or migrate substantial distances in biological fluids before targeting the recipient cell. Indeed, EVs have been found in urine, peripheral blood, saliva, breast milk, tears, and even cerebrospinal fluid (CSF), shuttling precise biological messages [20,21,22,23]. 

After shedding or being released from the parental cell, EVs can travel long distances until they reach the target cell and release their cargo. Current data suggest that the recipient cell influences the subsequent interaction mechanisms among EVs and itself [24]. Different strategies have been proposed for EV uptake via integrin adhesion or endocytosis, or through receptor–ligand interaction or target cell-dependent mechanisms still undefined [25]. The fate of the cargo and how it can exert multiple functions inside the recipient cell are still unsolved mysteries, even more so keeping in mind that the amount of cargo material is very exiguous [26]. EVs’ cargo primarily features lipids, proteins, and different nucleic acids, DNA, mRNA, and non-coding RNAs (ncRNAs), whose sorting mechanism is well sophisticated and reflects the donor cell [27]. Lipids such as sphingomielin, phospatidilserine, and cholesterol forming the EV membrane are the most representative elements of EVs. They preserve vesicle integrity, regulate recipient cell homeostasis, and concur in biogenesis and release processes [28]. The lipid content appears to be modulated in EVs with respect to the cell of origin that shows a reduction in several lipid classes rather than others and vice versa, while there are few works illustrating a selection of lipid species in EVs [28]. Instead, an asymmetric distribution between the two leaflets of EVs’ membranes is ascertained [29]. Regarding EVs’ protein composition, due to the huge amount of protein markers proposed to distinguish different subclasses of EVs, the MISEV 2018 guidelines include a list of protein categories belonging to non-cell/tissue-specific EVs (e.g., tetraspanins, integrins, ALIX, HSP70) and cell/tissue-specific EVs (e.g., CD9, CD90, CD45) or associated with specific intracellular compartments (e.g., histones for the nucleus, TOMM20 for mitochondria) [19]. As anticipated, EVs also carry specific proteins typical of the originated cell, located on their membrane and inside them. In particular, neural-derived EVs expose cell adhesion molecule 1 (L1CAM), GPI-anchored prion protein, and subunits of glutamate receptors labeling their origin [30]. Microglial EVs carry CD13 and monocarboxylate transporter 1, while oligodendrocyte-derived EVs contain myelin and associated lipids [31,32]. Astrocytes secrete EVs that move functional glutamate transporters and mitochondrial DNA (mtDNA) [33]. This peculiarity, plus their ability to cross the BBB, makes EVs eligible for CNS disease biomarker discovery. The scientific interest in EVs grew when genetic material, especially mRNAs and microRNAs, was also found belonging to EVs’ cargo. The observation that mRNA transcripts detected in EVs are deeply different from those of the parental cell testifies that the EV cargo packaging is not casual and could represent a sophisticated mechanism for genetic exchange between cells [34]. Similarly, a selection of miRNAs, attractive for their regulatory role in gene expression, seem to be exclusively sorted in EVs because they are undetectable in parental cells [34,35,36]. Interestingly, miRNAs represent the higher RNA component in EVs’ cargo, suggesting their critical role in the biology of the recipient cell [37]. Furthermore, deep sequencing analysis has shown that there are other types of small ncRNAs inside EVs, including tRNA fragments, repeat sequences, structural RNAs, vault RNA, Y RNA, and small interfering RNAs selectively enclosed in EVs [34,38]. Independently of cargo composition, it has now been established that it can affect the biology of the host cell in a physiological or pathological direction, meaning it could be hypothesized that proteins in EVs can trigger pathways of signal amplification and replication-competent RNA molecules [39]. As it has previously been mentioned and widely explained in the MISEV 2018 guidelines, the scientific community has not yet found a general consensus of a molecular signature able to discriminate different subclasses of EVs definitely, and there is no marker to distinguish them. For these reasons, we use the generic term EVs to refer to all vesicles, following the current recommendations of the International Society of Extracellular Vesicles (ISEV) [19].

## 3. Dual Role of EVs in MS: Neurodegenerative and Neuroprotective 

MS is the most common demyelinating disease of the CNS. It mainly affects young people during their third decade of life. Although the etiology remains unknown, autoreactive T and B cells that lead to an autoimmune response against myelin components drive the major event. Clinical manifestations are determined principally by inflammation, demyelination, and axonal degeneration, which are effectively the major pathologic hallmarks [40]. In MS, the communication between the immune system and CNS has been altered, and in this context, EVs could play a strategic part in both pathological and reparative mechanisms. EVs’ active role in the CNS is emphasized from evidence that most, if not all, cells belonging to the CNS secrete EVs, such as neural cells, glial cells, astrocytes, oligodendrocytes, and mast cells [14,41]. As already cited, MS represents the first neurological disorder where EVs were ascertained. Scolding et al., back in 1989, proved, for the first time, the presence of EVs in MS, demonstrating that recovery of oligodendrocytes from injury involved the release of EVs from the surface of cells, suggesting EVs’ contribution in myelin damage [16]. In fact, it seems that EVs play an inhibitory role in membrane biogenesis. Bakhti and colleagues reported, during CNS development, the inhibition of myelin sheath formation by oligodendrocyte-derived EVs via the Rho-associated coiled-coil protein kinase (ROCK) protein and actomyosin contractility [42]. EVs were released, and, consequently, their effect decreased significantly during incubation with a conditioned neuronal medium. It was suggested that neurons controlled the secretion of auto-inhibitory oligodendrocyte-derived EVs and coordinated myelin biogenesis [42].

The negative involvement of EVs in MS pathogenesis is not limited to myelin damage. EVs could dysregulate, mainly in acute phases of the disease, the immune response and, promoting cellular activation, amplify inflammation and contribute to BBB disruption [43]. 

Indeed, a study on experimental autoimmune encephalomyelitis (EAE), the murine model of MS, revealed that injection of microglial EVs into the brain of mice induces transendothelial recruitment of inflammatory cells to the injection site [44]. Moreover, the specific injection of microglia-derived EVs into mouse brains with EAE increased inflammation, worsening the disease [45]. Synaptic plasticity results in being deeply altered in MS [46]. Several recent pieces of evidence highlighted the ability of microglia- and astrocyte-derived EVs to influence the synaptic activity. After all, it has to be considered that neurons contain EVs and that synaptic activity is related to EVs’ fusion at the plasma membrane [47]. Gabrielli et al. showed that microglial EVs played a crucial role in the modulation of the inhibitory transmission by the direct stimulation of CB1R on target GABAergic neurons [48]. 

On the other hand, in MS pathogenesis, EVs could exert a neuroprotective role. It has been proposed that EVs play crucial roles in modulating the CNS synaptic plasticity, maintaining myelination, fixing damaged neurons [49,50]. In particular, EVs could act with positive behavior on synaptic activity. In Goldie et al.’s work, depolarized neurons released EVs enriched with synaptic plasticity-associated protein MAP1b [37]. Another study by Antonucci et al. identified that microglia- and astrocytes-derived EVs affect synaptic activity with the involvement of neuronal sphingosine [51]. Further evidence about EVs contributing to synaptic plasticity comes from the presence, in their cargo, of proteins implicated in synaptic button formation, such as the α-amino-3-hydroxy-5-methylisoxazole-4-propionic acid (AMPA) receptor components and the trafficking protein Evi/Wntless [47,52]. 

Not only proteins but also specific EV-derived RNAs have been suggested to absolve several functions in the plasticity processes. Recently, mRNA in association with the activity-regulated cytoskeleton-associated protein (Arc), a master regulator of synaptic plasticity, has been found enclosed in neuron-derived EVs [53]. The above-mentioned Goldie group found a selective enrichment and depletion of specific miRNAs in neurites, where a subset of these miRNAs was recovered in EVs, supporting their regulatory role in the neural plasticity process [37]. Instead, glial-derived EVs seem to regulate, in particular, the presynaptic activity affecting both excitatory and inhibitory neurotransmission [47]. It has been demonstrated that EVs are also involved also in the myelin biogenesis process. Indeed, it was shown that oligodendrocyte-derived EVs possess myelin proteins and specific RNA for the promotion of myelination processes [41]. Moreover, astrocyte-derived EVs were demonstrated to be enriched in synapsin 1 and could promote the growth, survival, and differentiation of nerve cells during development [54]. Taken together, this evidence supports an active role of EVs not only in myelination but also in oligodendrocyte and neuronal growth. 

If the exact contribution of EVs’ cargo in the pathogenesis of MS is yet to be clarified, there is, instead, a strong correlation between the levels of EVs and the phase of the disease (acute versus stable). In particular, Verderio et al. demonstrated the presence of a higher concentration of myeloid EVs in the CSF of MS patients in the acute phase of the disease. Similarly, increases in CSF myeloid EVs have also been detected in patients with clinically isolated syndrome (CIS). Moreover, EV levels correlated with gadolinium-enhancing MRI lesions, the clinical index of acute-phase MS, and some authors demonstrated that levels of myeloid EVs could represent a biomarker of neuroinflammation in MS, able to distinguish patients in the acute phase from the remission phase [44].

Several independent studies determined higher levels of EVs in MS compared with controls. Some works focused on plasmatic EVs of endothelial origin (EEVs) and related them to the acute phase of the disease, assuming that they promoted transendothelial migration of inflammatory cells and exacerbated the demyelination process [15,45,55]. Other works also extended the investigation to platelet-derived EVs, and lymphocyte- and monocyte-derived EVs [17]. 

The innovative approach of Mallardi and colleagues further confirmed what Verderio et al. previously determined [44], highlighting that a significant increase in plasmatic EVs (pEVs) could distinguish CIS and MS patients from controls [56].

Finally, a very recent work from Dalla Costa and coworkers further emphasized the prognostic role of EVs in CIS patients. Indeed, they reported that myeloid EVs are increased in the CSF of CIS patients compared to controls, and their concentration was related to a shorter time to evidence of disease activity (EDA) after the first demyelinating event [57].

## 4. EVs’ Cargo: Reservoir of Potential Biomarkers for MS

Recently, many studies focused on the determination of EVs’ cargo in MS as a biomarker (Table 1). 

EVs are particularly attractive in the challenging field of biomarker research in MS because they originate from a variety of cell sources, including cells of the CNS. Importantly, the existence of a correlation between EVs’ contents and the pathological conditions of the parental tissue, as with their well-protected cargo, can be easily assessed in biological fluids such as serum, plasma, urine, tears, and CSF, making them an ideal source of disease biomarkers. 

It has already been established that various elements inside EVs can influence the biology and immunity of cells. Among the different bioactive elements in EVs include proteins, lipids, and nucleic acids, mostly ncRNAs. Thus, the next subsections will illustrate recent findings about the relationship linking MS and EVs based on their different molecular contents.

### 4.1. EV-Derived Non-Coding RNAs

At present, the significance of the RNA content in EVs is advocated by the research community. MicroRNAs represent an EV RNA biotype more extensively studied and characterized. Alterations in miRNA profiles in MS have been demonstrated in relation to the clinical course, inflammatory processes, and treatment response. MicroRNAs regulate the expression, in health and disease, of protein-coding genes mainly causing inhibition of the target gene(s). 

Their relationship with disease status has been explained in two different works performed on serum-derived EV microRNAs, from MS patients, analyzed through NGS technology. Ebrahimkhani et al. found a cluster of nine miRNAs (miR-15b-5p, miR-23a-3p, miR-223-3p, miR-374a-5p, miR-30b-5p, miR-433-3p, miR-485-3p, miR-342-3p, miR-432-5p) differentially expressed in relapsing-remitting (RR) MS compared to progressive MS cases versus healthy controls (HC) [58], whereas Selmaj and colleagues showed that four serum-derived EV miRNAs (hsa-miR-122-5p, hsa-miR-196b-5p, hsa-miR-301a-3p, and hsa-miR-532-5p) were significantly reduced during relapse and radiological flare of RRMS patients [59]. 

Another study in RRMS patients found that EV miR-326, known to have a pathological role in MS, was significantly increased in those patients compared to HC [61]. In particular, miR-326’s in vitro targeting of E26 transcription factor 1 (Ets-1), a negative regulator of T cell differentiation, promoted Th-17 effector cells’ differentiation, contributing to neuroinflammation and myelin damage [75]. Importantly, miR-326 expression increased in PBMCs of patients in the relapsing phase compared with those in the remitting phase and HC. Thus, since miR-326 correlates with MS severity, it has been proposed as a biomarker of MS severity [76]. 

EV-associated miRNA profiling was performed to elucidate if there is an EV-derived miRNA signature depending on the IFN-β treatment in naïve, responder, and non-responder RRMS patients [60]. Array analysis of miRNAs extracted from serum EVs showed sixteen miRNAs modulated in treated patients versus naïve patients. In detail, fourteen were downregulated and two were upregulated, but they were mostly already differently involved in MS, including miR-23a, miR-15b, miR-223, miR-146a, let-7miRNAs, miR-451, and miR-26a [60,77]. Among these, miR-26a deserves attention. This miRNA is reported to modulate the Th17/Treg cell balance in EAE models by targeting IL-6 [78]. Additionally, miRNA-26a has been found to be upregulated in the relapsing phase of MS and showed the same expression pattern as miR-326 with in silico analysis [76]. Therefore, miR-26a, together with the above-mentioned miR-326, could be a reliable indicator of relapsing phases in MS patients.

Actually, in Manna’s work, since miRNA deregulation was confirmed only in treated patients and, in particular, in responders, the authors suggested that EV-derived miRNAs could be used to monitor the response to INF-β therapy [60]. Taken together, these results highlight clusters of miRNAs and not a specific miRNA alteration in relation to the stages of the disease [60]. 

Intriguingly, in vitro experiments of Prada et al. demonstrated that EVs released from inflammatory microglia move the miR-146a-5p cargo into neurons. This miRNA, specific to microglia and absent in hippocampal neurons, acts on the expression of presynaptic synaptotagmin1 (Syt1) and postsynaptic neuroligin1 (Nlg1), which participate in the dendritic spine formation impact on synaptic stability [63]. 

In addition to serum, plasma is also a rich source of EVs. Microarray analysis of plasma-derived EVs of HC and MS patients in the paper from Kimura et al. profiled the upregulation of four miRNAs; let-7i, miR-19b, miR-25, and miR-29a. Interestingly, let-7i, overexpressed in MS patients, was able to suppress Treg cell induction blocking the IGF1R/TGFBR1 pathway on naïve CD4+T cells, and in the group with major levels of EV-derived let-7i, the frequency of Treg cells was lower, contributing to the MS pathogenesis [62]. 

A very interesting source of EVs is represented by erythrocytes. They, lacking a nucleus, show a stable content of miRNAs. The availability of such peripheral source of EVs makes them a very interesting specimen in the study of EV-derived miRNAs in MS. A very recent study investigated, for the first time, the involvement of erythrocyte-derived EV miRNAs in MS [64]. Erythrocyte-derived EVs were purified ex vivo from isolated erythrocytes. This study highlighted that erythrocyte-derived EVs were selectively packaged and contained a majority of highly expressed miRNAs in red cells. In particular, hsa-miR-451a which is transferred from erythrocytes to endothelial cells by EVs, contributed to the loss of the integrity barrier in cerebral malaria, but it was also increased in MS patients’ plasma [64]. Actually, erythrocytes may have a role in MS due to their impaired antioxidant capacity and hemorheological changes that may contribute to BBB damage [64]. This peculiarity links cerebral malaria to MS. 

Myelin generation is a key process strictly related to MS. The possible role of mirR-219 in the myelination process has been studied in oligodendrocyte precursor cells (OPC). miR-219 poured by EVs into OPC increased their numbers and myelin production, inhibiting negative regulators of the myelination process [65]. The function of this miRNA is emblematic for MS because miR-219 is induced during OPC differentiation, playing a critical role in OPC maturation and maintenance of compact myelin, but it is depleted in MS lesions [65]. Furthermore, miR-219 could also be a potential biomarker of MS, supported by evidence where this miRNA was absent in the CSF of MS patients compared to HC, maybe because it was seized inside EVs [79]. 

Urinary EVs represent another interesting source of biomarkers thanks to their easy and non-invasive method of collection. Nevertheless, works of urinary EVs in MS are scarce. Indeed, only Singh’s group showed the potential usefulness of urine-derived EV miRNAs as a biomarker. Comparing EV miRNA profiles among the spinal cord (SP), plasma, and urine at the pre-onset, onset, and peak stages of EAE disease, the authors found an overexpression of EV-derived miR-155-5p during pre-onset [66]. This miRNA, also known as inflamma-miR, is a strong regulator of inflammation, modulating the autoimmune response in MS [80]. They also examined the effect of glatimer acetate (GA), usually used in MS therapy, on miRNA expression, showing, for the first time, that under treatment, especially in urinary EV miRNAs, expression was modulated, and miR-9-5p and miR-35-3p were significantly deregulated at the EAE peak stage. The authors suggested that urinary EVs could be used as a source of molecular biomarkers of disease progression and drug response. 

It has also been demonstrated that EVs carry the two polyomavirus JC (JCPyV) microRNAs, microRNA-J1-3p and microRNA-J1-5p, consistent with the high presence of JCPyV-DNA in positive MS samples [67]. Indeed, Giovannelli and collaborators detected, for the first time, both JCPyV miRNAs not only in blood cells but also in urinary and plasmatic EVs of patients at risk of progressive multifocal leukoencephalopathy (PML) versus HC. The reactivation of JCPyV, causing PML, represents a concrete risk of MS patients under natalizumab treatment [81]. Therefore, this finding could conceal a repression mechanism of viral replication during the asymptomatic phase of JCPyV. In this sense, the EV miRNA cargo could serve to discover earlier MS patients at risk of PML.

The involvement of other classes of EV non-coding RNAs in MS remains obscure. There is only one study pertaining to this topic and performed on the long non-coding RNA plasmacytoma variant translocation 1 (PVT1). PVT1 is recognized as an oncogene in cancer and downregulated in RRMS patients [82,83]. Wu and coworkers indirectly demonstrated that PVT1 was carried by EVs derived from M2 macrophages and inhibited Th17 cell proinflammatory response in EAE mice [68]. The treatment of EAE mice with M2-EVs was shown to reduce inflammation and to protect EAE mice, hypothesizing new therapy strategies.

### 4.2. EV-Derived Proteins

The EV protein cargo in MS remains rather unexplored. Galazka and colleagues, analyzing the EV protein content in the serum and CSF of MS patients, discovered, for the first time, the myelin oligodendrocyte glycoprotein (MOG), the most immunogenic myelin protein, expressed only on the surface of myelin sheaths and the oligodendrocyte membrane, strongly related to disease activity [69]. In serum-derived EVs, MOG was elevated in RRMS during relapse and in secondary progressive (SP) MS patients; in CSF, MOG levels were higher for all MS groups compared to controls without differences. Thus, it seems that the CSF MOG content in EVs reflects that in the serum. Theoretically, EVs might significantly propagate immunologic reactivity also outside the CNS. Furthermore, in order to exclude that MOG could derive from PBMCs rather than from the CNS, probably as a contaminant or as an unspecific protein, the authors stimulated serum PBMCs of patients and controls in vitro to release EVs. Despite the large amount of EVs generated, MOG cannot be detected in EVs from PBMC cultures.

A second study by Bhargava et al. is a pioneering work, and even if it has some limitations, it paves the way for further studies focusing on the role of TLRs (Toll-like receptors) inside serum EVs of RRMS patients. Basically, using an immune array to identify membrane proteins on circulating EVs, they found altered levels of TLR3 and TLR4 in MS subjects compared to controls [70]. Expressed in immune cells and in resident cells of the CNS, TLRs are responsible for pathogen recognition and host defense. Their function in MS human study is the opposite; TLR3 seems to exert a protective role, whereas TLR4 favors an inflammatory process [84]. Even though these research works have already demonstrated a role for these receptors in modulating MS and EAE, Bhargava’s work is the first analyzing the TLR content in EVs [70].

As it has previously been observed, pEVs could exacerbate inflammatory processes in MS patients [15,55]. This aspect could be related to the presence in pEVs’ cargo of fibrinogen that, in EAE murine models, is able to spontaneously induce relapses [71]. Indeed, in the same work, proteomics analysis on MS patients’ pEV cargo highlighted fibrinogen as an important fraction [71]. It has been ascertained that fibrinogen plays a key role in MS pathogenesis. This coagulation factor is the only one able to cross the BBB, persist in the CNS, and deposit by way of fibrin that incorporates fibronectin into the fibrin clot, recognized for its aggregation in MS lesions contributing to remyelination failure [85]. Fibrinogen in MS correlates with BBB disruption and inflammation activity, promotes new lesion formation, and inhibits tissue repair. On the other hand, the inhibition of fibrinogen expression in mutant mice protects from MS-like disease [86]. 

Other biological fluids such as CSF could uncover an affordable source of EV-derived protein biomarkers. Lee et al. analyzed the proteomic signature of 442 significant EV proteins extracted from the CSF of MS and neuromyelitis optica (NMO) patients. This analysis highlighted the significant presence of fibronectin, strictly related to MS, as above mentioned, and glial fibrillary acidic protein (GFAP), related to NMO, as well as different protein signatures able to discriminate MS from NMO. Thus, this study supports the hypothesis that EVs’ cargo not only could serve as a biomarker resource but also could assist in the correct identification of neuroinflammation disorders [22]. A pioneering study of proteomic profiling of EVs extracted from tears of MS patients for the first time demonstrated the presence of microglia-derived and neural-derived EVs [72]. Furthermore, the EV protein cargo from tears was about 70% identical to that isolated from CSF with similar and exclusive disease specific pathways compared to HC. This evidence supports the idea that tears could represent a valid source of biomarkers as much as CSF since eyes represent the natural extension of the brain, as proven by presence of oligoclonal bands (OCBs), used as a diagnostic tool for MS, in the tears of MS subjects [87,88]. 

### 4.3. EV-Derived Lipids

As it has previously been mentioned, the lipid component is more present in EVs, so much so that Wolf, in 1967, already defined them as lipid-rich particles [1]. As it has previously been explained, thanks to high-performance mass spectrometry platforms, it has already been reported that different species of lipids have emerged as characteristically enriched in the EV lipidome rather than that of the donor cell [28]. In addition to protecting the EV cargo from external injurious stimuli, lipids could also regulate the fundamental process of membrane fusion with the recipient cell [28]. Therefore, due to the critical role of lipid homeostasis in the CNS and lipids’ pivotal function in vesicles, EV-derived lipids could likely impact on neuroinflammation and CNS repair. In this regard, data from Moyano and colleagues showed a relevant increase in sulfatides in plasma EVs extracted from MS patients versus healthy controls, potentially representing a pathophysiology biomarker [73]. Sulfatides, together with cholesterols, phospholipids, and complex sphingolipids, represent the principal components of myelin sheaths, and even if their biological role is not totally understood, they are inevitably related, directly or indirectly, to MS [89]. Interestingly, EVs are enriched just with these classes of lipids that are essential for the remyelination event, assuming that EVs could be involved in this crucial process [28,90]. Indeed, Lombardi et al. provided, for the first time, proof that EV lipids are directly linked to myelin repair because OPC migration, the first fundamental stage of myelin regeneration, was stimulated by vesicular sphingosine 1 phosphate (S1P) released by microglial EVs [74].

## 5. EVs as New Therapeutic Approach in MS 

As it has previously been mentioned, EVs present on their surface-specific origin markers, and their cargo is finely shaped on the donor cell and on the target one; in addition, they can cross the BBB in both directions. For these peculiarities, EVs are appealing in the research of new therapeutic strategies apt to ideally rebuild the damaged myelin in MS patients (Table 2). 

The involvement of EVs in the myelination process emerged, for the first time, when oligodendrocyte-derived EVs were found to be enriched in myelin-related proteins such as myelin oligodendrocyte glycoprotein (MOG), proteolipid protein (PLP), myelin basic protein (MBP), and the 2′,3′-cyclic-nucleotide 3′-phosphodiesterase protein (CNP). Later, Frühbeis et al. also showed that the communication among oligodendrocytes and axons, essential for generating myelin, was mediated by EVs [32,96]. 

Actually, some studies emphasize the use of EVs as stimulators of the myelin regeneration process. When dendritic cell (DCs) cultures are stimulated with a low dosage of IFNγ, they release EVs which are preferentially taken up by oligodendrocytes responsible for myelination. Furthermore, the nasal administration of IFNγ-DCs-EVs enhanced myelin generation in vivo [97]. As it has already been pointed out, Pusic et al. [65] showed that serum EVs stimulate OPCs and their differentiation to produce mature myelin in physiological and demyelination conditions. The same work demonstrated that both young and aged rats exposed to environmental enrichment (EE; volitionally increased intellectual, social, and physical activity) released EVs with pro-myelinating effects. Furthermore, the nasal administration of serum EVs from young animals increased myelination in aging rats, assigning EVs a translational potential for their use in vivo [65]. 

Another way to counter myelin damage is to modulate the immune system, as current MS treatments do, to prevent the autoimmune attacks on myelin sheaths. In this line, EVs could be exploited as a shuttle of anti-inflammatory drugs, even intranasally, as demonstrated by Zhuang et al.’s work, where EVs containing curcumin or a Stat3 inhibitor reached the brain microglia, delaying EAE onset [91]. Another driver mechanism of demyelinating diseases is the neurodegeneration caused by axon disruption. Mesenchymal stem cell (MSC)-derived EVs seem to behave as peripheral immunomodulators, reducing inflammation and promoting neuroprotection, angiogenesis, and neurological function in traumatic brain injury models, paving the way for EVs’ therapeutic use for those pathologies such as MS where neuroprotection should be strengthened [98]. Moreover, MSCs are emerging as a promising tool in MS treatment [99,100,101]. In this regard, a myelin regenerating function is ascribed to EVs derived from MSCs proposed from different works. Injecting EVs extracted from placental MSCs in EAE mouse models, Clark and colleagues demonstrated that EVs could stimulate OPC differentiation, promoting new myelin formation [92]. Interestingly, intravenous administration of EVs derived from MSCs of human adipose tissue (hAdMSCs) had beneficial effects on a primary progressive MS murine model, ameliorating motor disability, reducing brain atrophy, and promoting remyelination as well as regulating neuroinflammation [93]. The authors of this study hypothesized that EVs from hAdMSC administration could contribute to fixing CNS damage, also restoring the myelination process [93]. Moreover, hAdMSC administration after brain injury is a validated therapeutic approach in mouse models because it promotes natural repairing processes [102,103]. Further, in EAE models, intravenous administration of EVs derived from hAdMSCs showed therapeutic implications, reducing T cell proliferation, leukocyte infiltration, and demyelination, even if the authors concluded that the effect of hAdMSCs on Treg cells was more appreciable than that of the respective EVs [94]. Farinazzo et al.’s work appears in contrast regarding EVs isolated from adipose stem cells (EV-ASCs) administrated intravenously on an EAE mouse model [95]. They demonstrated a protective effect of EV-ASCs only on EAE before disease onset but not on established EAE [95]. Despite this discrepancy, investigation on the potential use of EVs derived from MSCs for treating MS is worth pursuing as the cell-free alternative to MSC-based therapeutics.

It is also interesting to evaluate EVs as a diagnostic tool of staging disease. In the work of Sáenz-Cuesta and collaborators, they observed that after 5 h of fingolimod (FGM) administration, a marked increase in the EV concentration followed by a rapid and critical change of their miRNA cargo is present. This was an unexpected result because FGM, an analogue of natural sphingosine, should inhibit vesicular trafficking [104]. Furthermore, after the first dose of FGM, the inhibitory activity on lymphocytes of free EVs was reduced compared to EVs before FGM treatment. This evidence suggests that briefly after its administration, FGM regulates the release and immune activity of EVs in MS patients, making EVs suitable as biomarkers for early treatment monitoring [105].

## 6. Conclusions

The ever-growing increase in scientific articles in the EV field on neurological diseases, including MS, is doubtlessly related to: (1) their ability to cross the BBB in both directions; (2) the possibility to investigate them in all biological fluids, i.e., the potential use of peripheral fluids such as serum or plasma which overtake the invasiveness and cost of diagnostic and prognostic tools; (3) their content reflecting one of the donor cells and being well encased inside them; (4) the fine recognition mechanism, which is not totally understood, of target cells; (5) the presence on their surface of molecular markers almost completely specific to the donor cell; and (6) the possibility to use EVs as a biomarker reservoir also in the early phases of the disease. On the other hand, their application in clinical practice faced many barriers because there are still many technical issues to solve. The pre-analytical phase is characterized by many and variable factors affecting EV isolation and recovery, first of all, the type of matrix considered (serum, plasma, CSF, urine, etc.), the processing steps (processing time, type of tubes used, transport, etc.), and storage (fresh, frozen, temperature, etc.). As it is known, biological fluids show different biophysical and biochemical properties that have to be taken into consideration; serum and plasma are a rich source of EVs but, at the same time, are enriched with lipoproteins that co-precipitate with them, requiring adequate attention in the isolation step. On the contrary, CSF shows fewer EVs, but it represents a naturally depleted source of most contaminants. The second consideration is about the isolation methods to separate and concentrate them. They should be chosen based on the downstream applications, the type of matrix, and the purity degree, considering that the more purity required, the less the yield will be. Thus, due to the lack of standardization in the isolation protocols and for the extreme heterogeneity of MS disease, it is difficult to compare the results of different groups working on EVs and validate them in a clinical setting. Furthermore, the literature on EVs in MS is less abundant than other diseases, such as cancer or aging-related diseases. Nevertheless, it is worth betting on them considering that themselves and their cargo show most of the main credentials to be ideal biomarkers such as early visibility, easy accessibility, and origin specificity. 

However, it is hard to define what molecular nature the ideal biomarker should have. At present the most investigated biomarkers are miRNAs and proteins. The cargo of dysregulated miRNAs can vary in EVs from different origins, and they are usually studied in clusters, meaning it is difficult, at the time being, to highlight a unique specific miRNA as a biomarker of MS. Proteins are a more stable specimen, and proteomic analysis seems to be a useful strategy to highlight promising biomarkers, but few studies are available in the literature. On the other hand, EVs seem to represent, at the same time, one of the causes contributing to inflammatory progression. Due to this, analysis of their cargo could reveal not only new biomarkers of disease stages and targets for future treatments but also new landscapes on the MS pathophysiology. 

## Figures and Tables

**Table 1:** Molecular EVs’ cargo detected in MS patients, and in in vivo and in vitro models.

EVs Biological Source	EV Cargo	References
	**Non-coding RNAs**	
Serum	miR-15b-5p, miR-23a-3p, miR-223-3p, miR-374a-5p, miR-30b-5p, miR-433-3p, miR-485-3p, mir-342-3p, miR-432-5p	Ebrahimkhani et al. [58]
Serum	miR-122-5p, miR-196b-5p, miR-301a-3p, miR-532-5p	Selmaj et al. [59]
Serum	miR-23a, miR-15b, miR-223, miR-146a, mir-451, miR-26a, let7 family	Manna et al. [60]
T cell cultures	miR-326	Azimi et al. [61]
Plasma	miR-25, miR-19b, miR-29a, let7i	Kimura et al. [62]
Activated microglia	miR-146a-5p	Prada et al. [63]
Erythrocyte	miR-451a	Groen et al. [64]
Oligodendrocyte progenitor cells (OPC)	miR-219	Pusic et al. [65]
Urine of EAE models	miR-155-5p, miR-9-5p, miR-35-3p	Singh et al. [66]
Urine	miR-J1-3p and miR-J1-5p of JCPyV	Giovanelli et al. [67]
M2 macrophage of EAE models	lncRNA PVT1	Wu et al. [68]
	**Proteins**	
Serum and CSF	MOG	Galazka et al. [69]
Serum	TLR3 TLR4	Bhargava et al. [70]
Plasma of EAE models	Fibrinogen	Willis et al. [71]
CSF	Fibronectin GFAP	Lee et al. [22]
Tears and CSF	Integrin signaling events; PI3K signaling; EGF receptor (ErbB1) signaling pathway; ErbB receptor signaling network; IFNγ pathway; LKB1 signaling events; PDGF receptor signaling network; RNA polymerase I chain elongation; S1P1 pathway; signaling events mediated by VEGFR1 and VEGFR2; TNFα/NFkB; TRAIL signaling pathway	Pieragostino et al. [72]
	**Lipids**	
Plasma of MS pts	Sulfatides	Moyano et al. [73]
OPC of LPC mouse models	S1P	Lombardi et al. [74]

ncRNAs: non-coding RNAs; RRMS: relapsing-remitting multiple sclerosis; PTS: patients; OPC: oligodendrocyte precursor cell; EAE: experimental autoimmune encephalomyelitis; CSF: cerebrospinal fluid; SPMS: secondary progressive multiple sclerosis; NMO: neuromyelitis optica; LPC: lysolecithin; JCPyV: polyomavirus JC; PVT1: plasmacytoma variant translocation 1; MOG: myelin oligodendrocyte glycoprotein; TLR3 and TLR4: Toll-like receptors 3 and 4; GFAP: glial fibrillary acidic protein; S1P: sphingosine 1 phosphate.

**Table 2:** Therapeutic applications of EVs in MS.

Source of EVs	Effect	References
EVs from DCs	Nasal administration of IFNγ-DCs-EVs enhanced myelin generation in vivo	Pusic et al. [55]
EVs from young EE rats	Nasal administration of serum EVs from young animals increased myelination in aging rats	Pusic et al. [65]
EVs from glioblastoma cell line (GL26)	Nasal administration of EVs containing curcumin or Stat3 inhibitor reached brain microglia, delaying EAE	Zhuang et al. [91]
EVs from pMCs	Injection in EAE models of EV pMSCs stimulated OPC differentiation, promoting new myelin formation	Clark et al. [92]
EVs from hAdMCs	Intravenous administration of EVs from hAdMSCs in a primary progressive MS murine model ameliorated motor disability, reduced brain atrophy, and promoted remyelination, regulating neuroinflammation	Laso-Garcìa et al. [93]
EVs from hAdMCs	Intravenous administration of EVs from hAdMSCs reduced T cell proliferation, leukocyte infiltration, and demyelination	Jafarinia et al. [94]
EVs from ASCs	Intravenous administration of EV-ASCs in EAE model had a protective effect before EAE onset, but not on established EAE	Farinazzo et al. [95]

DC: dendritic cells; EE: environmental enrichment; MSCs: mesenchymal stem cells; hAdMSCs: human adipose mesenchymal stem cells; ASCs: adipose stem cells; PPMS: primary progressive multiple sclerosis; EAE: experimental autoimmune encephalomyelitis.

## Data Availability

Not applicable.
